# Assessment of Electronic Nicotine Delivery Systems With Cigarette Use and Self-reported Wheezing in the US Adult Population

**DOI:** 10.1001/jamanetworkopen.2023.6247

**Published:** 2023-04-03

**Authors:** Luz M. Sánchez-Romero, Irina Bondarenko, Marie Knoll, Jana L. Hirschtick, Steven Cook, Nancy L. Fleischer, David T. Levy

**Affiliations:** 1 Department of Oncology, Georgetown University, Washington, DC; 2 Department of Epidemiology, University of Michigan, Ann Arbor

## Abstract

**Importance:**

The prevalence of electronic nicotine delivery systems (ENDS) use has increased, leading to a concern about their respiratory health outcomes. It is unclear whether ENDS use increases the risk of wheezing, a common symptom of respiratory conditions.

**Objective:**

To provide an analysis of the longitudinal association between ENDS and cigarette use and self-reported wheezing among US adults.

**Design, Setting, and Participants:**

The US nationally representative Population Assessment of Tobacco and Health (PATH) Study was used. Longitudinal data for adults 18 years or older from wave 1 (2013-2014) to wave 5 (2018-2019) were analyzed. Data were analyzed from August 2021 to January 2023.

**Main Outcomes and Measures:**

The prevalence of self-reported wheezing (waves 2-5) was estimated for 6 strata of tobacco product use (never cigarette and noncurrent ENDS use, never cigarette and current ENDS use, current cigarette and noncurrent ENDS use, current cigarette and current ENDS use, former cigarette and noncurrent ENDS use, and former cigarette and current ENDS use). A generalized estimating equations framework evaluated the association between cigarette and ENDS use and self-reported wheezing at the subsequent wave. An interaction term between cigarette and ENDS use was added to estimate the association between joint cigarette and ENDS use categories and the association of ENDS within strata of cigarette use.

**Results:**

The analytical sample consisted of 17 075 US adults with a mean (SD) age of 45.4 (17) years, of whom 8922 (51%) were female and 10 242 (66%) were Non-Hispanic White. When compared with never cigarette and noncurrent ENDS use, the greatest association in reporting wheezing was for current cigarette and current ENDS use (adjusted odds ratio [AOR], 3.26; 95% CI, 2.82-3.77), which was similar to current cigarette and noncurrent ENDS use (AOR, 3.20; 95% CI, 2.91-3.51) and substantially greater than former cigarette and current ENDS use (AOR, 1.94; 95% CI, 1.57-2.41). Associations were small and not statistically significant for the odds of self-reported wheezing among never cigarette and current ENDS use when compared with never cigarette and noncurrent ENDS use (AOR, 1.20; 95% CI, 0.83-1.72), and for odds of wheezing and current cigarette and current ENDS use when compared with current cigarette and noncurrent ENDS use (AOR, 1.02; 95% CI, 0.91-1.15).

**Conclusions and Relevance:**

In this cohort study, exclusive ENDS use was not associated with an increase in the risk of self-reported wheezing. However, a small increase in risk between ENDS use and wheezing was reported by individuals who use cigarettes. This study adds to the literature about the potential health effects associated with ENDS use.

## Key Points

**Question:**

What is the association between cigarettes, electronic nicotine delivery systems (ENDS) use, and self-reported wheezing symptoms among the US adult population?

**Findings:**

Using 2013 to 2019 data from a cohort study of 17 075 participants in the US, this study found that ENDS use is not associated with increased odds of reporting wheezing independent of cigarette smoking.

**Meaning:**

These findings suggest ENDS use may not be solely responsible for increasing the odds of reporting wheezing, but it is associated with increased risk in the current and former cigarette smoking population.

## Teaser

This cohort study evaluates the association between electronic nicotine delivery systems, cigarette use, and self-reported wheezing in a population of US adults.

## Introduction

The use of electronic nicotine delivery systems (ENDS) has been increasing worldwide.^1,2^ Since their introduction to the US market in 2007, ENDS use has increased across all age groups, especially among young adults.^1,2,3,4,5^ The increase in popularity of ENDS products has been accompanied by a growing concern about their toxic effects and downstream health outcomes.^6^ The public health impact, for adults in particular, will in part depend on the health effects of ENDS relative to cigarettes, not only in terms of the respiratory effects of exclusive ENDS use^7^ but also the synergistic health risk when ENDS and other tobacco products are combined.^8,9^

Smoking (cigarette use) causes significant changes in the respiratory tract through various mechanisms.^6^ Physiological responses include excess production of mucus, reduction of the cilia movement, inflammation, and narrowing of the airways,^10^ which result in various respiratory symptoms such as coughing, phlegm, and dyspnea.^11^ Smoking has also been associated with wheezing, described as a whistling or high-pitched lung sound, that is a common respiratory symptom for several respiratory conditions, such as asthma and chronic obstructive pulmonary disease (COPD). Aside from the personal risks cigarette use carries, several studies have also found that even secondhand exposure to cigarette smoke can induce respiratory symptoms such as cough, sputum, and wheezing, especially among children.^12,13,14^

While several studies have associated smoking with wheezing,^15,16,17,18^ it is unclear whether ENDS use increases the risk of wheezing. The respiratory effects potentially depend not only on ENDS’ toxic effects but also on the individual’s smoking history.^6,19^ The role of ENDS use in wheezing is of particular interest because wheezing is an acute condition reflecting a more short-term impact than chronic conditions like COPD. However, literature on the association between ENDS use and wheezing is limited, with most of the evidence from cross-sectional studies using the first 2 waves of the US Population Assessment of Tobacco and Health (PATH) Study.^20,21,22^ Two studies using the PATH adult data found that ENDS exclusive and dual (ie, cigarettes in conjunction with ENDS use) use were both associated with wheezing, with dual use riskier than using either product alone.^20,22^ However, cross-sectional analyses do not allow researchers to disentangle the time ordering of ENDS use and wheezing. This is important given that adults who smoke cigarettes may switch to ENDS products after experiencing respiratory health symptoms.^23^

The purpose of this study is to provide a longitudinal analysis of the association between ENDS and cigarette use and self-reported wheezing symptoms among US adults using the PATH Study’s cohort data from waves 1 through 5. To our knowledge, this is the first study to use 5 waves of PATH to longitudinally assess the association between ENDS and wheezing across different cigarette use categories.

## Methods

We used cohort data from waves 1 (September 2013 to December 2014) to 5 (December 2018 to November 2019) of the PATH Study, a large nationally representative population-based study of tobacco use and health in the US.^24^ This study was approved by the institutional review board at the University of Michigan and follows the Strengthening the Reporting of Observational Studies in Epidemiology (STROBE) reporting guideline. PATH Study adult respondents provided written informed consent.

This study includes participants in PATH waves 1 through 5 who were 18 years and older at baseline and had complete sociodemographic and health history data at wave 1 (ie, baseline). Our analytical sample consisted of participants which constituted 90% of the adult cohort who participated in all 5 waves. Our total sample included observations across all waves with self-reported wheezing at follow-up. Analytical sample sizes for each wave were dependent on the number of individuals with complete data for self-reported wheezing and cigarette and ENDS use and baseline covariates.

### Wheezing Symptoms

Wheezing is a subjective complaint rather than a diagnosis. Wheezing was evaluated in the adult population 18 years and older in PATH using follow-up data from waves 2 (October 2014 to October 2015) to 5. Wheezing (yes or no) was defined according to the question: “Have you had wheezing or whistling in the chest in the past 12 months?”

### ENDS and Cigarette Use Exposure

To ensure that the cigarette and ENDS exposure did not occur after self-reported wheezing, we evaluated the association between product use at wave *t - 1* with self-reported wheezing at wave *t*. Thus, ENDS and cigarette use data were obtained from wave 1 to wave 4. The respondents were classified as current established ENDS users (yes or no) if they reported fairly regularly using ENDS, defined by the question, “Have you ever used electronic nicotine products fairly regularly” and if they reported current every day or some days use. Respondents were also stratified by their cigarette use behavior (ie, never, former, and current). Current cigarette smoking was defined as established use (having smoked 100 or more lifetime cigarettes) and currently smoking every day or some days. Former cigarette use was defined as established use but not currently smoking every day or some days. If a participant had not smoked 100 or more lifetime cigarettes, they were assigned to the never cigarette use category.

To study the association of joint categories of cigarette and ENDS use, we classified the population into 6 categories according to a combination of their smoking status (never, former, or current) and current established ENDS use: never cigarette and noncurrent ENDS user; never cigarette and current ENDS user; current cigarette and noncurrent ENDS user; current cigarette and current ENDS user; former cigarette and noncurrent ENDS user; and former cigarette and current ENDS user. A description of the survey questions used for this classification can be found in eTable 1 in Supplement 1.

### Covariates

We incorporated the following sociodemographic covariates as controls using baseline values (wave 1): age (as a continuous variable), sex (male or female), race and ethnicity (non-Hispanic White, non-Hispanic Black, Hispanic, non-Hispanic other [non-Hispanic American Indian/Alaskan Native, Asian, Native Hawaiian/Pacific Islander, and multiracial individuals]), and household annual income in $US ($50 000 or less, $50 000-$100 000, or $100 000 or more) as categorical variables. Disproportionate disparities in tobacco-related respiratory diseases have been observed in racial and ethnic minorities. Therefore, we decided to adjust our analyses by race and ethnicity to account for the influence of this risk factor into the outcome. We also adjusted the analyses for self-reported history of any respiratory disease (yes or no), obesity (body mass index ≥30; yes or no; body mass index is calculated as weight in kilograms divided by height in meters squared), and measures of secondhand smoke exposure at baseline to account for factors associated with wheezing and tobacco use. Secondhand smoke exposure was categorized into 3 groups: (none, 0-7 hours, or 7 or more hours per week) according to the response to: “During the past 7 days, about how many hours were you around others who were smoking? Include time in your home, in a car, at work, or outdoors.”

### Statistical Analysis

Descriptive statistics were first calculated for sociodemographic characteristics and factors associated with risk at baseline. We also calculated the prevalence and SE for all the product use categories across waves 1 to 4. We also estimated the prevalence and SE of self-reported wheezing at any point in time for the overall sample and each of the 6 categories of product use across waves 2 to 5.

Generalized estimating equations (GEE) were used to evaluate the association between cigarette use and ENDS use and self-reported wheezing. The GEE method, developed by Liang and Zeger,^25^ takes into account the correlation of within-participant data when analyzing repeated measures with nonnormal response variables.

GEE were used to evaluate the association between cigarette use and ENDS use and self-reported wheezing. Statistical models at a significance threshold of *P* < 0.5 were considered statistically significant. We adjusted for sociodemographic variables at baseline (ie, age, wave, sex, race and ethnicity, history of respiratory disease, obesity, and secondhand smoking) while accounting for correlation between observations from the same respondents. Then, to estimate the association between ENDS use within the strata of cigarette use and wheezing we introduced an interaction term between cigarette and ENDS use.

For all the analyses, we estimated marginal logistic regression models with unstructured covariance and within-person correlation matrices and the binomial distribution of the dependent variable using the logit link function. Variances were computed using the balanced repeated replication method applying the replicate weights provided by the PATH study. For GEE analyses, we applied PROC GENMOD for each of 100 replicate weights and combined results using Fay adjustment set to 0.3 to increase estimate stability.

All analyses were conducted using SAS version 9.4 software (SAS Institute).^26^ Tests were based on weighted data using PATH sample wave 5 “all-wave” weights (including full-sample and 100 replicate weights). Data were analyzed from August 2021 to January 2023.

## Results

Our analytical sample included 17 075 individuals with a mean (SD) age of 45.4 (17.0) years, of whom 8922 (51.5%) were women, 10 242 (66.0%) were non-Hispanic White, 5956 (49.5%) reported nonexposure to second hand-smoking and 3195 (16.4%) reported a history of respiratory disease. Descriptive characteristics of the analytical sample at baseline are presented in eTable 2 in Supplement 1.

Table 1 shows the distribution of product use and self-reported wheezing by wave for the analytical sample. Prevalence of wheezing of the entire sample slightly declined from 16.4% at wave 2 to 14.3% at wave 5, with the highest frequency observed at wave 2. The prevalence of current cigarette use remained stable across the 4 waves varying between 18.0% and 19.0%, with 23.0% of the sample reporting cigarette use of at least 1 year in waves 1 to 4. In comparison, 6.0% of adults reported ENDS use at least once across follow-up, but for many participants ENDS use behavior remain stable from wave to wave, with current ENDS use at 2.6%, 3.5%, 3.3%, and 3.0% in waves 1, 2, 3 and 4, respectively. Only 0.3% of adults reported exclusive ENDS use, while most adults who used ENDS report current or former established cigarette use.

**Table 1.** Sample Characteristics of the Population Assessment of Tobacco and Health Study at Each Wave of Data Collection

Characteristics	% (SE)				
	Wave 1 (2013-2014)	Wave 2 (2014-2015)	Wave 3 (2015-2016)	Wave 4 (2016-2018)	Wave 5 (2018-2019)
Unweighted No.^a^	16 987	16 951	17 000	17 031	NA
Wheezing, yes^b^	NA	16.4 (0.35)	16.2 (0.37)	15.3 (0.36)	14.3 (0.35)
ENDS use					
Current	2.6 (0.12)	3.5 (0.16)	3.3 (0.13)	3.0 (0.13)	NA
Noncurrent	97.5 (0.12)	96.5 (0.16)	96.7 (0.13)	97.0 (0.13)	NA
Cigarette use					
Never	61.8 (0.61)	58.7 (0.64)	57.1 (0.65)	56.3 (0.66)	NA
Former	20.1 (0.48)	22.6 (0.49)	24.3 (0.50)	25.3 (0.53)	NA
Current	18.1 (0.31)	18.7 (0.32)	18.6 (0.37)	18.3 (0.36)	NA
Categories of product use					
Never cigarette users					
Noncurrent ENDS users	61.8 (0.62)	58.6 (0.64)	57.1 (0.66)	56.3 (0.66)	NA
Current ENDS users	0.3 (0.03)	0.3 (0.03)	0.3 (0.04)	0.3 (0.03)	NA
Current cigarette users					
Noncurrent ENDS users	16.2 (0.30)	16.5 (0.30)	16.7 (0.35)	16.7 (0.33)	NA
Current ENDS users	1.6 (0.09)	2.1 (0.11)	1.8 (0.09)	1.7 (0.10)	NA
Former cigarette users					
Noncurrent ENDS users	19.5 (0.47)	21.6 (0.48)	23.2 (0.47)	24.2 (0.50)	NA
Current ENDS users	0.6 (0.05)	1.0 (0.08)	1.1 (0.08)	1.1 (0.07)	NA

Abbreviations: ENDS, electronic nicotine delivery system; NA, not applicable.

^a^ This is an unmatched panel; thus, fluctuations could arise from actual changes in participants with complete data.

^b^ Prevalence and SE of self-reported wheezing was estimated from waves 2 to 5.

The Figure shows the prevalence of wheezing in the 6 categories of tobacco product use at the previous wave: never cigarette and noncurrent ENDS use, never cigarette and current ENDS use, current cigarette and noncurrent ENDS use, current cigarette and current ENDS use, former cigarette and noncurrent ENDS use, and former cigarette and current ENDS use. Prevalence of wheezing was highest among current cigarette and noncurrent ENDS use and current cigarette and current ENDS use, exceeding 30.0% across all waves. Self-reported wheezing was lowest for never cigarette and noncurrent ENDS use (decreasing from 10.3% in wave 2 to 8.8% in wave 5), followed by former cigarette and noncurrent ENDS use. Among the never cigarette and current ENDS use group, prevalence of wheezing varied from 24.0% in wave 2 to 13.1% in wave 5.

**Figure.**
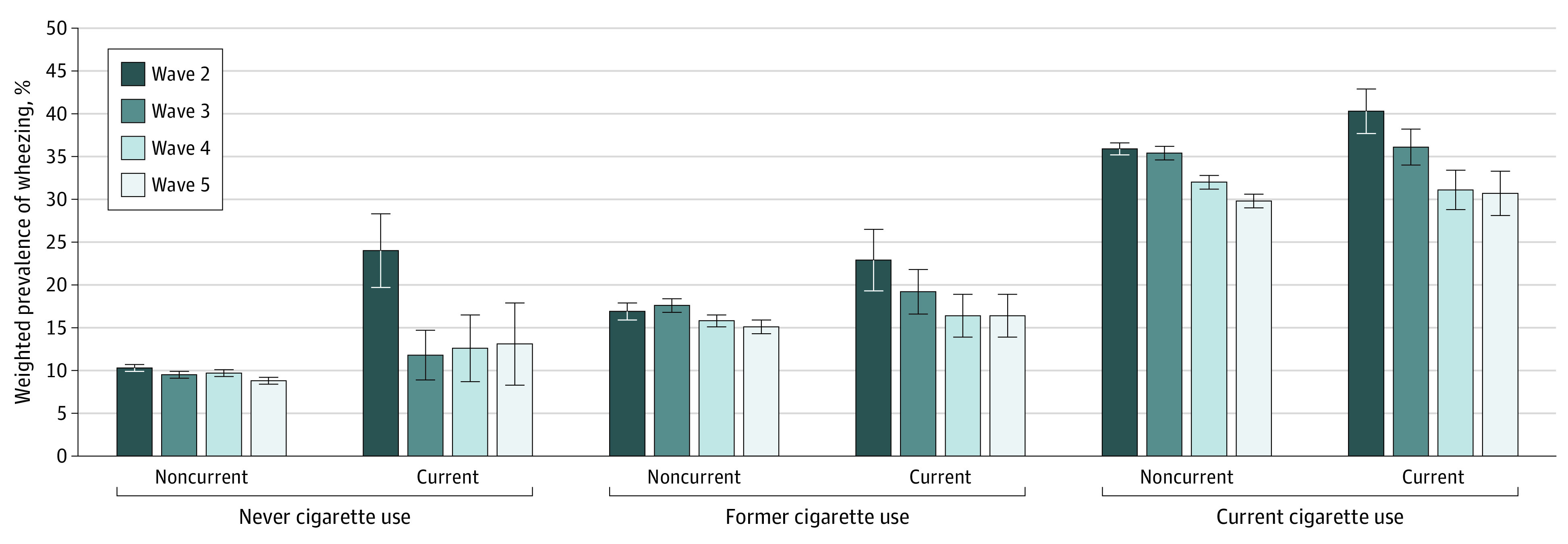
Prevalence of Self-reported Wheezing by Cigarette and Electronic Nicotine Delivery System Use at Previous Wave Error bars indicate SEs.

### Self-reported Wheezing and Baseline Characteristics

Table 2 presents the results of the logistic GEE model for the multivariable association between ENDS use (current and noncurrent) and cigarette smoking (current, former, or never) with self-reported wheezing. Overall, ENDS use was not associated with self-reporting wheezing at the follow-up wave (adjusted odds ratio [AOR], 1.09; 95% CI, 0.98-1.21), but current (AOR, 3.15; 95% CI, 2.87-3.46) and former (AOR, 1.50; 95% CI, 1.33-1.68) cigarette use showed a positive association.

**Table 2.** Results of the Logistic Generalized Estimated Equation Models for Self-reported Wheeze and ENDS and Cigarette Use at the Previous Wave^a^

Variable	AOR (95% CI)	*P* value
Current ENDS use		
No current	1 [Reference]	NA
Current	1.09 (0.98-1.21)	.12
Cigarette use		
Never	1 [Reference]	NA
Current	3.15 (2.87-3.46)	<.001
Former	1.50 (1.33-1.68)	<.001
Age at baseline	1.10 (1.07-1.13)	<.001
Sex		
Male	1 [Reference]	NA
Female	0.89 (0.81-0.99)	.03
Race and ethnicity		
Hispanic	0.64 (0.55-0.74)	<.001
Non-Hispanic Black	0.73 (0.63-0.84)	<.001
Non-Hispanic White	1 [Reference]	NA
Non-Hispanic other^b^	0.82 (0.70-0.97)	.02
Income, $		
50 000 or less	1.40 (1.24-1.59)	<.001
50 000-100 000	1.14 (0.99-1.30)	.07
100 000 or more	1 [Reference]	NA
Respiratory disease ever		
No	1 [Reference]	NA
Yes	6.84 (6.24-7.49)	<.001
History of secondhand exposure, h/wk		
0	1 [Reference]	NA
<7	1.29 (1.12-1.48)	<.001
≥7	1.87 (1.62-2.16)	<.001
Obesity at baseline		
No	1 [Reference]	NA
Yes	1.59 (1.45-1.74)	<.001
Wave	0.93 (0.90-0.95)	<.001

Abbreviations: AOR, adjusted odds ratio; ENDS, electronic nicotine delivery system; NA, not applicable.

^a^ Generalized estimating equation model without interactions adjusted for sociodemographic variables at baseline: age, sex, race and ethnicity, history of respiratory disease, obesity, and secondhand smoking exposure and wave (continuous) while accounting for correlation between observations from the same participant.

^b^ Non-Hispanic other category includes non-Hispanic American Indian/Alaskan Native, Asian, Native Hawaiian/Pacific Islander, and multiracial individuals.

Multivariable associations between other variables and self-reported wheezing can also be found in Table 2. The odds of reporting wheezing were found to increase by 10.0% (AOR, 1.10; 95% CI, 1.07-1.13) with each decade of age at baseline, and by 59.0% (AOR, 1.59; 95% CI, 1.45-1.74) for adults with obesity, controlling for product use status. Non-Hispanic Black (AOR, 0.73; 95% CI, 0.63-0.84) and Hispanic (AOR, 0.64; 95% CI, 0.55-0.74) respondents had lower odds of wheezing than non-Hispanic White respondents. Compared with respondents who reported a household income of greater than $100 000, respondents with an income of less than $50 000 had higher odds (AOR, 1.40; 95% CI, 1.24-1.59) of reporting wheezing. Female participants had lower odds of self-report wheezing than males (AOR, 0.89; 95% CI, 0.81-0.99). Compared with adults without secondhand smoke exposure, adults with secondhand smoke exposure greater than 0 but less than 7 hours per week (AOR, 1.29; 95% CI, 1.12-1.48) and with 7 or more hours per week (AOR, 1.87; 95% CI, 1.62-2.16) had increased odds of self-reported wheezing. Finally, there was an association between self-reported wheezing and history of respiratory diseases observed (AOR, 6.84; 95% CI, 6.24-7.49). Results from a sensitivty analysis considering a time-lag effect on wheezing can be seen in eTable 3 in Supplement 1.

Table 3 and Table 4 present the odds ratios for the association between wheezing and the 6 categories of product use (never cigarette and noncurrent ENDS use, never cigarette and current ENDS use, current cigarette and noncurrent ENDS use, current cigarette and current ENDS use, former cigarette and noncurrent ENDS use, and former cigarette and current ENDS use), adjusted for the set of covariates listed in Table 2. Table 3 shows the odds ratio of self-reported wheezing by comparing product use category against the never cigarette and noncurrent ENDS use group. We found that never cigarette and current ENDS use was not associated with a significant increase in odds of self-reported wheezing (AOR, 1.20; 95% CI, 0.83-1.72) compared with never cigarette and noncurrent ENDS use. However, former cigarette and noncurrent ENDS use was associated with a 48.0% (AOR, 1.48; 95% CI, 1.32-1.67) increase in odds of reporting wheezing, and former cigarette and current ENDS use with 94.0% (AOR, 1.94; 95% CI, 1.57-2.41) increase in odds. The largest increase in odds of self-reported wheezing was when respondents reported current cigarette use. The odds of self-reported wheezing for current cigarette and noncurrent ENDS users (AOR, 3.20; 95% CI, 2.91-3.51) was nearly the same as the odds of the current cigarette and ENDS group (AOR, 3.26; 95% CI, 2.82-3.77).

**Table 3.** Adjusted Odds Ratios for Association Between Cigarette-ENDS Use and Self-reported Wheezing Compared With Never Cigarette and Noncurrent ENDS Group^a^

Cigarette use	Noncurrent ENDS use, AOR (95% CI)	*P* value	Current ENDS use, AOR (95% CI)	*P* value
Never	1 [Reference]	NA	1.20 (0.83-1.72)	.32
Former	1.48 (1.32-1.67)	<.001	1.94 (1.57-2.41)	<.001
Current	3.20 (2.91-3.51)	<.001	3.26 (2.82-3.77)	<.001

Abbreviations: AOR, adjusted odds ratio; ENDS, electronic nicotine delivery system; NA, not applicable.

^a^ Association of wheezing and 6-level tobacco use at the previous wave adjusted per age at baseline, wave, sex, race and ethnicity, history of respiratory disease, obesity, and secondhand smoking.

**Table 4.** Adjusted Odds Ratios for Association Between Cigarette-ENDS Use and Self-reported Wheezing Within Strata of Cigarette Use^a^

Cigarette use	ENDS use, AOR (95% CI)		*P* value
	Noncurrent	Current	
Never	1 [Reference]	1.20 (0.83-1.72)	.32
Former	1 [Reference]	1.31 (1.08-1.58)	.008
Current	1 [Reference]	1.02 (0.91-1.15)	.73

Abbreviations: AOR, adjusted odds ratio; ENDS, electronic nicotine delivery system.

^a^ Association of wheezing and 6-level tobacco use at the previous wave adjusted per age at baseline, wave, sex, race and ethnicity, history of respiratory disease, obesity, and secondhand smoking.

By evaluating the association between ENDS use within the strata of cigarette use and wheezing (Table 4), we found that, within the former cigarette use category, current ENDS use was associated with a 31.0% (AOR, 1.31; 95% CI, 1.08-1.58) increase in odds of self-reported wheezing compared with no current ENDS use. There was a 20.0% increase in odds of wheezing associated with ENDS use within the never cigarette use category, but this increase was not statistically significant (AOR, 1.20; 95% CI, 0.83-1.72). No association was found between ENDS use and wheezing within the current cigarette use category (AOR, 1.02; 95% CI, 0.09-1.15). In addition, we conducted sensitivity analyses stratifying ENDS into 3 new categories, never, noncurrent (former), and current. We observed a statistically significant increase in self-reported wheezing among former cigarette and current ENDS users (AOR, 1.33; 95% CI, 1.08-1.63) compared with former cigarette and never ENDS users (eTable 4 and eTable 5 in Supplement 1).

## Discussion

Using US nationally representative PATH cohort data, we examined the association between ENDS use and cigarette use with self-reported wheezing among adults. First, we looked at ENDS and cigarette use as separate independent variables and observed no overall association between ENDS use and self-reported wheezing. Next, we combined cigarette and ENDS into a 6-category exposure variable and found that self-reported wheezing was most associated with current dual use of cigarettes and ENDS, followed by current cigarette and noncurrent ENDS use and by former cigarette and current ENDS use compared with never cigarette and noncurrent ENDS use.

The main findings from our analysis suggest that current ENDS use does not appear to increase the odds of self-reported wheezing independent of cigarette smoking. However, former cigarette use accompanied by current ENDS use was associated with an increased risk of reporting wheezing compared with former cigarette and noncurrent ENDS use. Similar results were observed when we conducted a sensitivity analyses stratifying ENDS use into 3 new categories: never, noncurrent (former), and current. Our sensitivity analysis, including 9 categories instead of 3, showed similar results. This suggests that aside from the lasting impact of cigarettes on adult respiratory health in the former use group, ENDS use could independently increase the odds of reporting wheezing. It may be that adults who used ENDS had higher pack-years before cessation or used ENDS due to an underlying case of wheezing compared with those who did not use ENDS.

Our study did not find an association between exclusive ENDS use and self-reported wheezing. Still, our other significant results are in line with those from several cross-sectional studies using single waves of PATH data, which found that current cigarette users or dual users have higher risks of self-reported wheezing than exclusive ENDS when compared with noncurrent ENDS use. This result is also supported by other longitudinal analyses^27,28,29^ and cross-sectional data,^30^ which by testing various biomarkers, determined that dual use resulted in greater toxicant exposure compared with using either product alone.

In our study, compared with noncurrent ENDS or never cigarette use, former cigarette and noncurrent ENDS use had a greater positive association with reporting wheezing, though never cigarette and current ENDS use did not, suggesting that there are lasting effects of cigarette smoking on respiratory symptoms even after quitting. This finding has been observed in other studies.^31,32^ Aherrera et al^33^ observed in a cohort study of 150 participants that exclusive ENDS use was associated with more wheezing than nonuse. However, after adjusting for sex, age, and former smoking status, this association was not statistically significant. Among adults who currently smoked cigarettes, we detected no association between ENDS use and wheezing. This suggests that ENDS use may not be responsible for increasing the odds of reporting wheezing in the current cigarette smoking population. This result is similar to findings in PATH cross-sectional studies that observed no significant differences in risk of wheezing between dual or polytobacco use and current cigarette smoking.^20,22^

### Strengths and Limitations

Our study has several strengths. We used nationally representative data which allows us to apply our findings to the entire population of US adults at baseline. The PATH survey is comprehensive in that it enabled us to adjust for many relevant covariates. Unlike cross-sectional studies, the longitudinal nature of this study allowed us to establish temporality between tobacco use and subsequent respiratory outcomes. In addition to covering more PATH waves than previous studies, our methods also considers participants’ dual use of products.

Despite these strengths, this study has several limitations that are important to note. All data were self-reported, which may lead to recall and social desirability bias, as well as potential unobserved confounding. The sample size of some of our cigarette and ENDS use categories could limit the ability to detect the true association. Additionally, data across waves contain information about ENDS products available at the time of the survey, meaning products from wave 1 and wave 5 are different and may not represent what is currently on the market. Phrasing of questions in the PATH study could result in misclassification of ENDS use. Wave 1 asks about “e-cigarettes” and waves 2 and 3 ask about “e-products.” Excluding participants who were missing data for variables of interest in any of the waves, in combination with the relatively low prevalence of ENDS users, limits interpretation and the power of observing individual outcomes of ENDS use in those who are dual users.

## Conclusions

Using data from a nationally representative cohort study between 2013 and 2019, our findings suggest that the use of ENDS does not appear to be an independent factor associated with risk for self-reported wheezing. However, a small increase in risk between ENDS use and wheezing was reported by individuals with a history of cigarette use. This study adds to the body of literature that investigates the potential harms and benefits associated with ENDS use compared with other tobacco products. Our results can help inform public health policy recommendations for ENDS use under different tobacco product user categories.

## Notes

Supplement 1. **eTable 1. **Categories of Tobacco Exposure Status **eTable 2. **Distribution of Baseline Covariates in Analytic Sample **eTable 3. **Results of the Logistic Generalized Estimated Equation (GEE) Models (Cross- Lagged and Ages) for Self-reported Wheeze and ENDS and Cigarette Use at the Previous Wave **eTable 4. **Results of the Logistic Generalized Estimated Equation (GEE) Models (for Self-Reported Wheeze and 3 Categories of ENDS at the Previous Wave Results From a Sensitivity Analysis **eTable 5. **Adjusted Odds Ratio for Association Between Cigarette-ENDS§ Use† 3 Categories and Self-Reported Wheezing Within Strata of Cigarette Use

Supplement 2.
Data Sharing Statement
