# Supplementary material for: Assessment of Electronic Nicotine Delivery Systems With Cigarette Use and Self-reported Wheezing in the US Adult Population
Source: JAMA Netw Open. 2023 Apr 3;6(4):e236247. doi: 10.1001/jamanetworkopen.2023.6247 (PMC10071334; doi:10.1001/jamanetworkopen.2023.6247)
Supplement: Supplement 1. — eTable 1. Categories of Tobacco Exposure Status eTable 2. Distribution of Baseline Covariates in Analytic Sample eTable 3. Results of the Logistic Generalized Estimated Equation (GEE) Models (Cross- Lagged and Ages) for Self-reported Wheeze and ENDS and Cigarette Use at the Previous Wave eTable 4. Results of the Logistic Generalized Estimated Equation (GEE) Models (for Self-Reported Wheeze and 3 Categories of ENDS at the Previous Wave Results From a Sensitivity Analysis eTable 5. Adjusted Odds Ratio for Association Between Cigarette-ENDS§ Use† 3 Categories and Self-Reported Wheezing Within Strata of Cigarette Use [file jamanetwopen-e236247-s001.pdf]

Supplemental Online Content

Sánchez-Romero LM, Bondarenko I, Knoll M, et al. Assessment of electronic nicotine delivery systems with cigarette use and self-reported wheezing in the US adult population. *JAMA Netw Open*. 2023;6(3):e236247. doi:10.1001/jamanetworkopen.2023.6247

eTable 1. Categories of Tobacco Exposure Status

eTable 2. Distribution of Baseline Covariates in Analytic Sample

eTable 3. Results of the Logistic Generalized Estimated Equation (GEE) Models (Cross-Lagged and Ages) for Self-Reported Wheeze and ENDS and Cigarette Use at the Previous Wave

eTable 4. Results of the Logistic Generalized Estimated Equation (GEE) Models (for Self-Reported Wheeze and 3 Categories of ENDS at the Previous Wave Results From a Sensitivity Analysis

eTable 5. Adjusted Odds Ratio for Association Between Cigarette-ENDS§ Use† 3 Categories and Self-Reported Wheezing Within Strata of Cigarette Use

This supplemental material has been provided by the authors to give readers additional information about their work.

© 2023 Sánchez-Romero LM et al. *JAMA Network Open.*

eTable 1. Categories of Tobacco Exposure Status

	Never cigarette user	Former cigarette user	Current cigarette user
**No Current ENDS[a] use**	Haven't smoked 100 cigarettes in lifetime.<br>Haven't used ENDS fairly regularly	Have smoked 100+_ cigarettes in lifetime<br>No current cigarette use. No current ENDS use	Have smoked 100+_ cigarettes in lifetime<br>Current cigarette use on some/every day<br>No current ENDS use
**Current ENDS use**	Haven't smoked 100 cigarettes in lifetime.<br>Current ENDS use on some/every day	Have smoked 100+_ cigarettes in lifetime<br>No current cigarette use. Current ENDS use on some/every day	Have smoked 100+_ cigarettes in lifetime.<br>Current cigarette use on some/every day<br>Current ENDS use on Some/Every day

[a]Electronic nicotine delivery systems

© 2023 Sánchez-Romero LM et al. *JAMA Network Open.*

eTable 2. Distribution of Baseline Covariates in Analytic Sample

Covariate		Unweighted N	Weighted % (SE)
**Age (mean)**		17075	45.4 (0.11)
**Sex**			
	Female	8922	51.5 (0.22)
	Male	8153	48.5 (0.22)
**Race/Ethnicity**			
	Hispanic	2939	14.8 (0.16)
	Non-Hispanic White	10242	66.0 (0.24)
	Non-Hispanic Black	2666	11.7 (0.16)
	Non-Hispanic other	1228	7.5 (0.14)
**Income**			
	<50K	7214	33.3 (0.57)
	50-100K	3903	23.1 (0.50)
	100K+	5958	43.7 (0.63)
**Exposure to Second-hand Smoking at Baseline**			
	None	5956	49.5 (0.72)
	1-7 hours/week	7006	35.2 (0.53)
	7+ hours/week	4113	15.3 (0.40)
**History of Respiratory Disease**			
	No	13880	83.6 (0.33)
	Yes	3195	16.4 (0.33)
**Obesity at Baseline**			
	No	11666	68.5 (0.51)
	Yes	5409	31.5 (0.51)

© 2023 Sánchez-Romero LM et al. *JAMA Network Open.*

eTable 3. Results of the Logistic Generalized Estimated Equation (GEE) Models (Cross- Lagged and ages) for Self-Reported Wheeze and ENDS[a] and Cigarette Use at the Previous Wave[b]

Variable	AOR (95% CI)-Cross Lagged<br>Waves 2-5	AOR (95% CI)-Lagged model<br>Wave 3-5
**Current ENDS Use (ref=No Current ENDS Use)**		
Current ENDS Use	1.09 (0.98,1.21)	1.01 (0.86,1.19)
**Cigarette Use (ref= Never cigarette user)**		
Current Cigarette User	3.15 (2.87,3.46)**	2.23 (2.03,2.45)**
Former Cigarette Use	1.50 (1.33,1.68)**	1.29 (1.15,1.45)**
**Age at Baseline**	1.10 (1.07,1.13)**	1.08 (1.05,1.10)**
**Gender (ref=Male)**		
Female	0.89 (0.81,0.99)*	0.92 (0.84,1.00)
**Race/Ethnicity (ref=Non-Hispanic White)**		
Hispanic	0.64 (0.55,0.74)**	0.77 (0.68,0.87)**
Non-Hispanic Black	0.73 (0.63,0.84)**	0.87 (0.78,0.98)*
Non-Hispanic Other[c]	0.82 (0.70,0.97)*	0.82 (0.72,0.94)*
**Income (USD) (ref=$100,000 or more)**		
50,000 or less	1.40 (1.24,1.59)**	1.25 (1.13,1.39)**
50,000-100,000	1.14 (0.99,1.30)	1.05 (0.93,1.17)
**Respiratory Disease Ever (ref=No)**		
Yes	6.84 (6.24,7.49)**	2.82 (2.54,3.13)**
**Obesity at baseline (ref=No)**		
Yes	1.59 (1.45,1.74)**	1.34 (1.25,1.44)**

[a]Electronic nicotine delivery system.
[b]GEE model without interactions adjusted for sociodemographic variables at baseline: age, sex, race/ethnicity, history of respiratory disease, obesity, and second-hand smoking exposure and wave (continuous) while accounting for correlation between observations from the same participant.
[c] Non-Hispanic Other category includes NH American Indian/Alaskan Native, Asian, Native Hawaiian/Pacific Islander, and multiracial individuals.
*p-value <0.05 ** p-value <0.001

© 2023 Sánchez-Romero LM et al. *JAMA Network Open.*

eTable 4. Results of the Logistic Generalized Estimated Equation (GEE) Models (for Self-Reported Wheeze and 3 categories of ENDS[a] at the Previous Wave[b] Results From a Sensitvity Analysis

Variable	AOR (95% CI)	P-value
**Current ENDS Use (ref=Never ENDS Use)**		
Current ENDS Use	1.13 (1.00,1.26)	0.05
Non-Current ENDS Use	1.12 (1.01,1.25)	0.03
**Cigarette Use (ref= Never cigarette user)**		
Current Cigarette User	3.06 (2.78,3.36)	<0.001
Former Cigarette Use	1.44 (1.28,1.62)	<0.001
**Age at Baseline**	1.11 (1.08,1.14)	<0.001
**Gender (ref=Male)**		
Female	0.89 (0.81,0.99)	0.03
**Race/Ethnicity (ref=Non-Hispanic White)**		
Hispanic	0.64 (0.55,0.73)	<0.001
Non-Hispanic Black	0.73 (0.64,0.85)	<0.001
Non-Hispanic Other[c]	0.82 (0.69,0.96)	0.02
**Income (USD) (ref=$100,000 or more)**		
50,000 or less	1.40 (1.24,1.59)	<0.001
50,000-100,000	1.13 (0.99,1.29)	0.08
**Respiratory Disease Ever (ref=No)**		
Yes	6.78 (6.18,7.44)	<0.001
**History of second-hand exposure (ref=0 hours/week)**		
<7 hours/week	1.28 (1.12,1.47)	<0.001
7+ hours/week	1.87 (1.61,2.16)	<0.001
**Obesity at baseline (ref=No)**		
Yes	1.59 (1.45,1.74)	<0.001

[a]Electronic nicotine delivery system.
[b]GEE model without interactions adjusted for sociodemographic variables at baseline: age, sex, race/ethnicity, history of respiratory disease, obesity, and second-hand smoking exposure and wave (continuous) while accounting for correlation between observations from the same participant.
[c] Non-Hispanic Other category includes NH American Indian/Alaskan Native, Asian, Native Hawaiian/Pacific Islander, and multiracial individuals.

© 2023 Sánchez-Romero LM et al. *JAMA Network Open.*

eTable 5. Adjusted Odds Ratio for Association Between Cigarette-ENDS[§] Use[†] 3 Categories and Self-Reported Wheezing Within Strata of Cigarette Use

[§]Electronic nicotine delivery system

Cigarette Use	Never ENDS use	Current ENDS use<br>AOR (95% CI)	P-value	Non-Current ENDS use<br>AOR (95% CI)	P-value
**Never user**	**Reference**	1.28 (0.89,1.85)	0.19	1.43 (1.00,2.04)	0.05
**Former user**	**Reference**	1.33 (1.08,1.63)	0.01	1.20 (0.98,1.48)	0.08
**Current user**	**Reference**	1.05 (0.92,1.20)	0.45	1.07 (0.95,1.21)	0.27

[†]Association of wheezing and 6-level tobacco use at the previous wave adjusted per age at baseline, wave, sex, race/ethnicity, history of respiratory disease, obesity, and second-hand smoking.
[*]p-value <0.05
[**]p-value <0.001

© 2023 Sánchez-Romero LM et al. *JAMA Network Open.*
