# Supplementary material for: Assessment of Electronic Nicotine Delivery Systems With Cigarette Use and Self-reported Wheezing in the US Adult Population
Source: JAMA Netw Open. 2023 Apr 3;6(4):e236247. doi: 10.1001/jamanetworkopen.2023.6247 (PMC10071334; doi:10.1001/jamanetworkopen.2023.6247)
Supplement: Supplement 2. — Data Sharing Statement [file jamanetwopen-e236247-s002.pdf]

Data Sharing Statement

Sánchez-Romero. Assessment of Electronic Nicotine Delivery Systems With Cigarette Use and Self-reported Wheezing in the US Adult Population. *JAMA Netw Open*. Published April 03, 2023. doi:10.1001/jamanetworkopen.2023.6247

Data
Data available: Yes
Data types: Deidentified participant data
How to access data: https://pathstudyinfo.nih.gov/
When available: With publication

Supporting Documents
Document types: None

Additional Information
Who can access the data: Researchers whose proposed use of the data has been approved
Types of analyses: For research purposes
Mechanisms of data availability: after approval of a proposal and with a signed data access agreement
